# Structural Modification of the Antidepressant Mianserin Suggests That Its Anti-inflammatory Activity May Be Independent of 5-Hydroxytryptamine Receptors

**DOI:** 10.3389/fimmu.2019.01167

**Published:** 2019-05-24

**Authors:** Sandra Sacre, Albert Jaxa-Chamiec, Caroline M. R. Low, Giselle Chamberlain, Cathy Tralau-Stewart

**Affiliations:** ^1^Brighton and Sussex Medical School, University of Sussex, Brighton, United Kingdom; ^2^Drug Discovery Centre, Imperial College of Science, Technology, and Medicine, London, United Kingdom

**Keywords:** inflammation, toll-like receptor, rheumatoid arthritis, antidepressant, mianserin, macrophage, 5-hydroxytryptamine, tumor necrosis factor

## Abstract

Antidepressants are increasingly recognized to have anti-inflammatory properties in addition to their ability to treat major depressive disorders. To explore if engagement of 5-hydroxytryptamine (5-HT) receptors was required for the anti-inflammatory effect of the tetracyclic antidepressant mianserin, a series of structural derivatives were generated with the aim of reducing 5-HT receptor binding. Primary human peripheral blood mononuclear cells were used to screen for anti-inflammatory activity. The lead compound demonstrated a significant loss in 5-HT receptor binding, as assessed by non-selective 5-HT binding of radiolabelled serotonin in rat cerebral cortex. However, it retained the ability to inhibit endosomal toll-like receptor 8 signaling in primary human macrophages and spontaneous cytokine production from human rheumatoid synovial tissue equivalent to that previously observed for mianserin. These data demonstrate that the anti-inflammatory mechanism of mianserin may be independent of 5-HT receptor activity. This research offers new insights into the mechanism and structural requirements for the anti-inflammatory action of mianserin.

## Introduction

Antidepressants have been used for decades in the treatment of major depressive disorders (MDD). These drugs consist of several classes, each with varying mechanisms of action that modulate monoaminergic signaling (1). In recent years, it has been increasingly acknowledged that many antidepressants have an additional anti-inflammatory activity, that may contribute toward the clinical benefit of these drugs when treating MDD (2). The pathological link between inflammation and MDD has been established in several ways. Patients with MDD have elevated levels of serum pro-inflammatory cytokines, which are restored to levels similar to healthy controls in patients that respond to treatment (3). MDD can also occur as a co-morbidity of many chronic inflammatory diseases such as rheumatoid arthritis (RA), where cytokines are systemically elevated (4). Furthermore, data from a meta-analysis study, suggests that the use of anti-cytokine therapies such as anti-TNF, improves depressive symptoms in patients being treated for chronic inflammatory diseases including rheumatoid arthritis (5). Consequently, there has been much interest in the development of anti-inflammatory drugs to treat MDD.

The anti-inflammatory activity of antidepressants has been confirmed in classical models of inflammation. For example, the selective serotonin reuptake inhibitor fluoxetine reduces inflammation in the rat carrageenan induced paw edema model and a murine collagen induced arthritis model (6, 7). In addition, the atypical antidepressant mianserin which acts via a different mechanism, as an antagonist/inverse agonist at a range of aminergic G-protein coupled receptors (GPCRs) and inhibits noradrenaline uptake, also ameliorates disease progression in the murine collagen induced arthritis model and is effective in the rat B1 kinin receptor induced paw edema model (8, 9). Mianserin, fluoxetine and citalopram can also inhibit spontaneous cytokine production in human synovial membrane cultures from rheumatoid arthritis patients (7, 10).

This anti-inflammatory effect may in part be mediated by their ability to suppress signaling downstream of the toll-like receptor (TLR) family of innate immune receptors. Mianserin, fluoxetine and citalopram selectively inhibit cytokine production induced by a subset of the TLRs expressed within the endosome, in primary human M-CSF derived macrophages, B-lymphocytes, synovial fibroblasts and murine bone marrow derived macrophages (7, 10). Whereas, other antidepressants such as nortriptyline and amitriptyline have been shown to inhibit TLR4 in rat mixed glial cultures (11).

Despite understanding that these drugs have immunomodulatory properties, the precise mechanism remains undefined and it is still unclear whether this is a downstream consequence of modulating monoaminergic signaling or a distinct property of these compounds. Our previous study in primary human macrophages and rheumatoid synovial tissue required concentrations far higher than those reported for 5-HT receptor binding, indicating that the anti-inflammatory activity may be an off target effect, distinct from the role of these drugs in monoaminergic signaling (10, 12). However, several studies have suggested that the anti-inflammatory activity may be partially mediated by an increased availability of 5-HT elevating cAMP and Ca^2+^ through activation of monoamine receptors (13, 14).

A better understanding of the anti-inflammatory mechanism of antidepressants will be necessary before developing new anti-inflammatory drugs to treat MDD and may provide a new approach for treating chronic inflammatory diseases. In order to verify if the anti-inflammatory activity of mianserin was independent of 5-HT receptor binding, we designed a series of derivatives of mianserin with the aim of reducing the affinity for 5-HT receptors. Compounds were initially screened for their immunomodulatory activity. Following these experiments, one compound was identified which, despite showing a dramatically reduced affinity for 5-HT receptors, still retained full anti-inflammatory activity in human macrophages and human rheumatoid synovial membrane cultures.

## Materials and Methods

### Reagents

Cell culture reagents used were Penicillin-Streptomycin, RPMI 1640 obtained from Cambrex (Belgium) and fetal bovine serum from Life Technologies Ltd (UK). The TLR ligands used were chloroform extracted *Escherichia coli (E.coli)* lipopolysaccharide (LPS) and resiquimod (R-848) from Invivogen (USA). Flagellin (purified) and Pam_3_cys-ser(lys)_4_.3HCl (Pam3) were from Alexis (UK). Mianserin hydrochloride was purchased from Sequoia Research Products (Pangbourne, UK). The structural derivatives of mianserin were synthesized by Oxygen Healthcare Research Pvt. Ltd. (Ahmedabad, India), the synthesis steps are provided in the Supplemental Information. Macrophage colony stimulating factor (M-CSF) was purchased from eBioscience (USA). Dimethyl sulfoxide (DMSO) and Hank's balanced salt solution (HBSS) without calium chloride and magnesium sulfate were purchased from (Sigma, UK). Percoll Plus was purchased from GE Healthcare (Bucks, UK), PBS citrate from Paris Anticorps (France) and lympholyte-H from CedarLane (Ontario, Canada).

### Design Hypothesis for the Structural Modification of Mianserin

**Figure 1:**
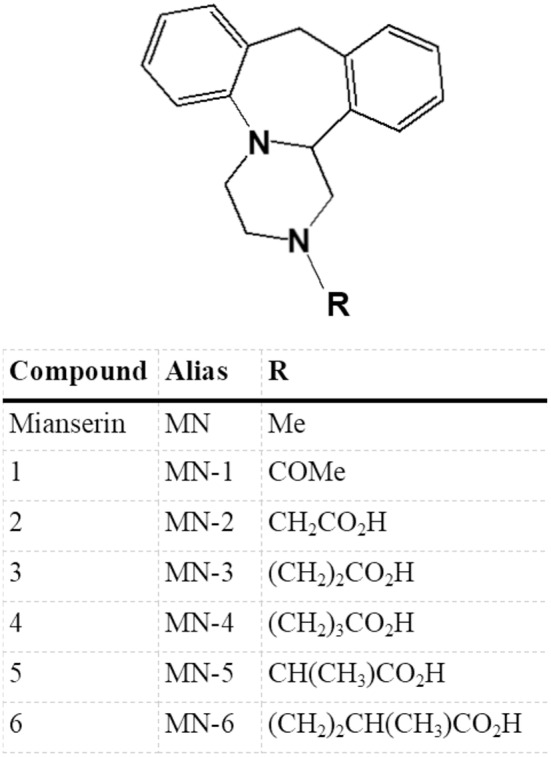
Structural derivatives of mianserin. A series of compounds were generated based on mianserin with modification of the R group with the aim of retaining inhibition of the anti-inflammatory action of mianserin (MN) but with greatly reduced activity at 5-hydroxytryptamine (5-HT) receptors. In the case of MN-1 the secondary amine was acetylated, for MN2-6 the methyl group of mianserin was removed and replaced with polar functionalities.

Mianserin is a tetracyclic antidepressant whose primary activity is as an antagonist/inverse agonist of a range of aminergic GPCRs. Literature reports include 5-HT, α-adrenergic and histamine H1 receptor activity, but mianserin is also reported to inhibit noradrenaline uptake (Table 1). To explore the requirement of 5-HT receptor binding for the immunomodulatory activity of mianserin, compounds were designed to bias activity in favor of mianserin's anti-inflammatory effects. In our previous study, mianserin was observed to inhibit endosomal TLR induced cytokine production (10). As TLRs are not known to preferentially bind simple amines, the structure was modified in ways that were predicted to be unlikely to bind to the monoamine GPCRs. Although a wide range of mianserin derivatives have previously been described, all retain the N-methyl group, which is one of the primary pharmacophore elements for aminergic receptor binding (16). It has long been hypothesized that central nervous system drugs share common binding features, principally aromatic groups and a basic amine. In the case of MN-1 the secondary amine was acetylated. For the other compounds, the methyl group was removed, the basic nitrogen retained and a polar group attached via an alkyl linker (MN-2, 3, 4, 5, and 6). The intention was to retain inhibition of TLR-induced cytokines but greatly reduce activity at 5-HT receptors (Figure 1).

**Table 1:** Reported activity of mianserin at human and rat G-protein coupled receptors.

**Receptor**	**Activity**	**Median pKi human (no. data points)**	**Median pKi rat (no. data points)**
5-HT_1A_		6.3 (1)	6.3 (1)
5-HT_2A_	Antagonist	8.37 (7)	8.6 (5)
5-HT_2B_	Antagonist	8.6 (2)	7.3 (1)
5-HT_2C_	Inverse agonist	8.36 (9)	9.13 (2)
5-HT_6_	Antagonist	7.3 (3)	7.4 (1)
5-HT_7_	Antagonist	7.43 (4)	7.4 (3)
α_1A_-adrenoreceptor	Antagonist	7.6 (1)	7.04 (1)
α_1B_-adrenoreceptor	Antagonist	7.4 (2)	7.18 (1)
α_1D_-adrenoreceptor	Antagonist	7.44 (2)	No data available
Histamine receptor H_1_	Antagonist	9.26 (2)	No data available
Noradrenaline transporter	Uptake inhibitor	7.6 (1)	7.59 (3)

*Activity data are from guidetopharmacology.org accessed on 31/1/19. IUPHAR/BPS Guide to pharmacology website version 2019.1, release date 30/1/19 (15). 5-HT, 5-hydroxytryptamine*.

### Cell Culture

Leukocyte cones from blood donors, were purchased from NHS Blood and Transplant (Tooting, UK). Written informed consent was obtained from the donors by NHS Blood and Transplant and the study was approved by Brighton and Sussex Medical School Research Governance and Ethics Committee. Peripheral blood mononuclear cells (PBMCs) were isolated from the cones by Lympholyte-H centrifugation. Briefly, cells were diluted to 40 mls with HBSS, then layered onto two tubes each containing 20 mls of Lympholyte-H and centrifuged for 25 min at 900 × g. The PBMC layer was then collected and washed twice in HBSS by centrifugation at 350 × g for 10 min. PBMCs were cultured at 37°C in a cell culture incubator with 5% CO_2_ and 21% O_2_. Cells were plated at 2 × 10^5^ cells/well in 96-well tissue culture plates (Corning, Amsterdam, The Netherlands) in Roswell Park Memorial Institute (RPMI) 1640 media containing 5% (v/v) FBS and 100 U/ml penicillin/streptomycin or used for the isolation of monocytes. Peripheral blood monocytes were obtained by density centrifugation of PBMCs on an iso-osmotic Percoll gradient. Iso-osmotoic percoll was achieved by adding 1 vol 1.5 M NaCl to 9 vol of Percoll Plus. The gradient was then prepared by mixing 1:1 v/v isosmotic percoll with 1x phosphate buffered saline (PBS) citrate. Briefly, cells were diluted to 20 mls with HBSS, then layered onto a tube containing 20 mls of iso-osmotic Percoll and centrifuged for 15 min at 900 × g. The monocyte layer was then collected and washed twice in HBSS by centrifugation at 350 × g for 10 min.

Immediately after isolation, monocytes were cultured at 37°C in a cell culture incubator with 5% CO_2_ and 21% O_2_. Cells were plated at 2 × 10^5^ cells/well in 96-well tissue culture plate in RPMI 1640 containing 5% (v/v) FBS and 100 U/ml penicillin/streptomycin. Macrophages were derived from monocytes after differentiation for 4 days in RPMI containing 5% (v/v) FCS, 100 U/ml penicillin-streptomycin) and 100 ng/ml M-CSF. Macrophages were then pre-incubated with media alone, MN-1 or DMSO (vehicle control) for 30 min, then stimulated with 10 ng/ml LPS, 100 ng/ml PAM3cys, 100 ng/ml Flagellin or 1 μg/ml R-848 for 6 h after which the supernatants were collected.

RA synovial membrane cells were isolated as previously described (17, 18), from joint synovium removed from donors during elective joint replacement surgery. All patients gave written informed consent and the study was approved by the South East Coast—Brighton and Sussex research ethics committee (10/H1107/27). Immediately after isolation cells were cultured at 4 × 10^4^ cells per well in a 384 well tissue culture plate (Corning, Amsterdam, The Netherlands) in RPMI containing 5% (v/v) FBS and 100 units/ml Penicillin/streptomycin. Cells were then incubated in the media alone or media containing a range of concentrations of MN-1 for 24 h. Cell viability was assessed after all experiments by the 3-[4,5 dimethylthiazol-2-yl]-2,5-diphenyl-tetrazolium bromide (MTT) assay (Sigma) (19). Briefly, cells were incubated with 100 μl of 0.5 mg/ml MTT in complete cell culture medium for 4 h at 37°C in a cell culture incubator with 5% CO_2_ and 21% O_2_ to allow formazan crystal formation in metabolically active cells. To dissolve the crystals, 100 μl of MTT lysis buffer (10% SDS and 0.01 M HCl) was then added for 3 h or until all crystals had dissolved. Absorbance was read at 570 nM on a Biotek synergy HT spectrophotometric ELISA plate reader and analyzed using the associated Gen5 software (Biotek, Bedfordshire, UK).

### ELISA (Enzyme-Linked Immunosorbent Assay)

Sandwich ELISAs were employed to measure tumor necrosis factor (TNF) (BD Pharmingen, UK) and interleukin (IL)-1β (R&D systems, Abingdon, UK); TNF capture antibody (551220), TNF biotinylated antibody (554511), IL-1β capture antibody (MAB601), and biotinylated IL-1β antibody (BAF201) were used according to the manufacturer's instructions. Recombinant TNF and IL-1β were purchased from PeproTech (London, UK). Streptavidin HRP (DY998) was used at 1:400 dilution and 3,3′,5,5′-Tetramethylbenzidine microwell peroxidase substrate kit (KPL Inc., USA) was used according to the manufacturer's instructions. All incubation steps were for 1 h at room temperature, apart from the incubation of the standards and samples which was overnight at 4°C. Color formation was stopped using 0.16 M sulfuric acid. Absorbance was read on a Biotek synergy HT spectrophotometric ELISA plate reader and analyzed using the associated Gen5 software.

### Non Selective Serotonin (5-HT) Receptor Binding Assay

The affinity of MN and MN-1 toward 5-HT receptors was determined by Eurofins Scientific France (Poitiers, France) using radioligand binding techniques similar to those described by Peroutka et al. (20). A non-selective serotonin receptor binding assay was chosen due to mianserin having activity at several of the 5-HT receptors (Table 1). Briefly, membrane homogenates of cerebral cortex (170 μg protein) were incubated for 60 min at 37°C with 2.5 nM [^3^H]serotonin in the absence or presence of 10 μM mianserin or MN-1 in a buffer containing 50 mM Tris-HCl (pH 7.4), 4 mM CaCl_2_, 10 μM pargyline and 1 g/l ascorbic acid. Nonspecific binding is determined in the presence of 10 μM serotonin.

Following incubation, the samples were filtered rapidly under vacuum through glass fiber filters (GF/B, Packard, Perkin Elmer, UK) pre-soaked with 0.1% bovine serum albumin and rinsed several times with ice-cold 50 mM Tris-HCl using a 96-sample cell harvester (Unifilter, Packard, Perkin Elmer, UK). The filters are dried then counted for radioactivity in a scintillation counter (Topcount, Packard, Perkin Elmer, UK) using a scintillation cocktail (Microscint 0, Packard, Perkin Elmer, UK).

### Statistical Methods

Mean, standard deviation (SD), standard error of the mean (SEM), and statistical significance were calculated using GraphPad version 3 (GraphPad Software Inc., USA). For statistical analysis of multiple means a one way ANOVA with a 95% confidence interval, followed by a Dunnett's multiple comparisons test was used. For the comparison of 2 means, a two tailed *t*-test of paired data was used with a 95% confidence interval. SEM was used for pooled experimental data whilst SD was used in graphs showing representative experiments. ^****^p < 0.0001, ^**^p < 0.01, and ^*^p < 0.05.

## Results

### MN-1 Inhibited TLR8 Induced TNF Production in Human PBMCs Which Was Equivalent to Mianserin

**Figure 2:**
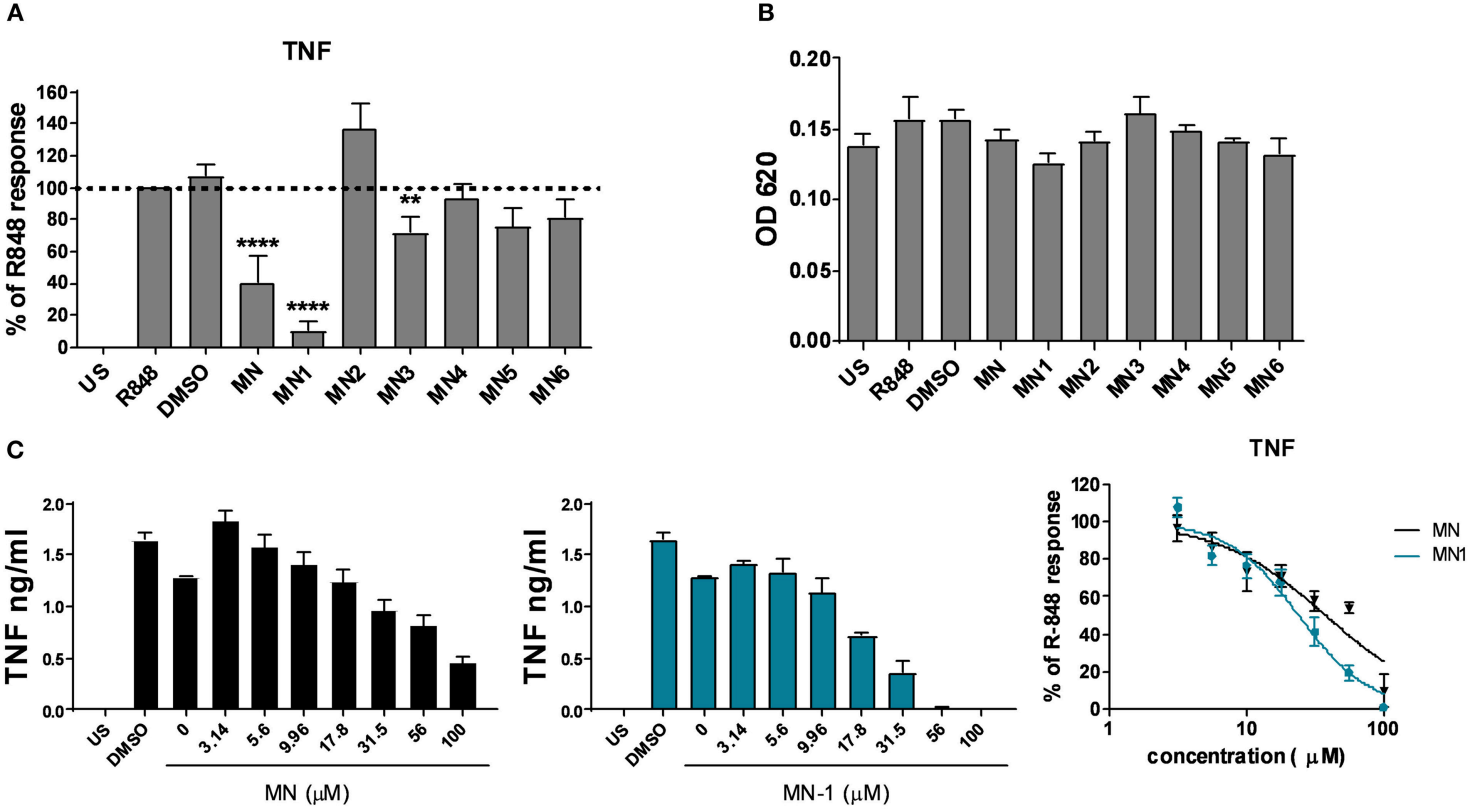
MN-1 inhibited toll-like receptor 8 induced tumor necrosis factor (TNF) production in PBMCs that was equivalent to mianserin. **(A)** Peripheral blood mononuclear cells (PBMCs) were pre-incubated with media alone unstimulated (US), dimethyl sulfoxide (DMSO) (vehicle control) or 80 μM of mianserin (MN), MN-1, MN-2, MN-3, MN-4, MN-5 or MN-6 for 30 min before stimulation with 2 μg/ml R-848 for 18 h. The data are shown as a percentage of the TNF production in R-848 stimulated cells from 4 to 9 independent donors. A one way ANOVA with a 95% confidence interval was used to test significance, followed by a Dunnett's multiple comparisons test compared to the cells stimulated with R-848 alone (^****^p < 0.0001 and ^**^p < 0.01). **(B)** Cell viability was assessed by a 3-[4,5 dimethylthiazol-2-yl]-2,5-diphenyl-tetrazolium bromide assay. Data are representative of 3 separate experiments performed in triplicate in 3 unrelated donors. **(C)** PBMCs were pre-incubated with media alone, DMSO or a quarter log titration of MN or MN-1 for 18 h. Data are shown for a representative donor and as pooled data from three donors shown as a percentage of the TNF production from R-848 stimulated cells in the absence of MN or MN-1. Standard error of the mean was used for pooled experimental data whilst standard deviation was used in graphs showing representative experiments.

As we had previously demonstrated an anti-inflammatory effect of mianserin on endosomal TLR signaling, a human PBMC assay was used to screen for activity of the structural derivatives against TLR8 activation. R-848 was used as a ligand for TLR8; although this is a dual ligand for TLR7, it is thought to act solely through TLR8 in human monocytes and macrophages as these cells do not produce TNF in response to TLR7 ligands (10). At a set concentration of 80 μM only mianserin, MN-1 and MN-3 were able to significantly inhibit the production of TNF upon stimulation with the TLR7/8 ligand R-848 (Figure 2A). However, MN-3 only produced a weak inhibition that was variable between donors. Cell viability was assessed using the MTT assay and found to be unaffected (Figure 2B). The concentration of 80 μM was chosen as a sub-optimal inhibitory concentration for the initial screening, to permit detection of compounds with improved inhibitory activity. This had been determined during a previous study where 100 μM mianserin produced an almost complete inhibition of TLR8 (10). As MN-1 produced the strongest inhibitory activity, it was chosen for further study. A dose response curve was generated from TLR8 activated human PBMCs using a quarter log titration to compare the EC_50_ of MN-1 with mianserin. DMSO was used as a loading control at an equivalent amount to the 100 μM dose of MN-1. When the data was pooled from three independent experiments using blood from three separate donors, the calculated EC_50_ for mianserin and MN-1 was 37.91 μM and 24.09 μM respectively (Figure 2C). Dose response curves were not generated for the other compounds, as this would have required using significantly elevated concentrations leading to cell toxicity.

### 5-HT Receptor Binding Activity of MN-1 Was Significantly Reduced in Comparison to Mianserin

To determine if MN-1 was still capable of binding 5-HT receptors at levels comparable to that of mianserin, the affinity of MN-1 for 5-HT receptors (non-selective) was evaluated in rat cerebral cortex in a radioligand binding assay. A non-selective serotonin receptor binding assay was used due to mianserin being reported to have activity at several 5-HT receptors (Table 1). The activity of mianserin at 5-HT receptors is also reported to be in the nM range, so a concentration of 10 μM mianserin and MN-1 was chosen for this assay to ensure these inhibitors would be in excess of what is required to block 5-HT binding (21). The rat cerebral cortex is a useful model for these experiments, as mianserin has a similar pKi on human and rat 5-HT receptors (Table 1). At 10 μM mianserin inhibited [^3^H] serotonin binding to 5-HT receptors with an average inhibition of 94.5%, whereas MN-1 showed a significantly decreased effect with an average inhibition of only 30% (Table 2).

**Table 2:** MN-1 has decreased 5-HT receptor binding compared to mianserin.

**Compound**	**% Inhibition of control specific serotonin binding**			
	**Experiment 1**	**Experiment 2**	**Mean**	**Standard deviation**
Mianserin	88.9	100	94.5	7.8
MN-1	27.1	32.8	30	4

*10 μM Mianserin and 10 μM MN-1 were measured for their ability to inhibit non selective 5-hydroxytryptamine (5-HT) binding of [^3^H] radiolabelled serotonin in rat cerebral cortex. The results are expressed as a percent inhibition of the control radioligand specific binding*.

### MN-1 Retained Selectivity for Endosomal TLRs in M-CSF Derived Primary Human Macrophages

**Figure 3:**
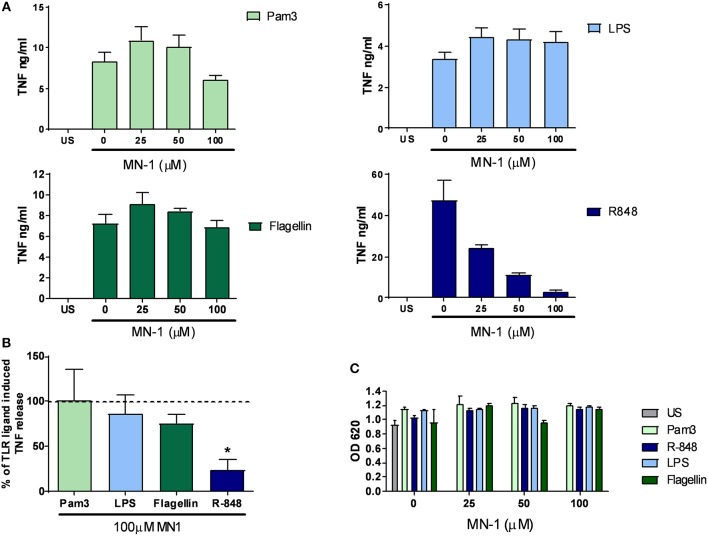
MN-1 inhibited toll-like receptor (TLR) 8 but not TLR1/2, 4 or 5 signaling in monocyte colony stimulating factor (M-CSF) derived macrophages. M-CSF derived macrophages were pre-incubated for 30 min with MN-1 before stimulation with 100 ng/ml Pam_3_cys-ser(lys)_4_.3HCl (Pam3) (TLR1/2), 10 ng/ml lipopolysaccharide (LPS) (TLR4), 10 ng/ml Flagellin (TLR5), 1 μg/ml resiquimod (R-848) (TLR8) or left unstimulated (US) for 6 h. **(A)** Data are representative of 3 separate experiments performed in triplicate in 3 unrelated donors. **(B)** TNF production in the presence of 100 μM MN-1 is shown as a percentage of the TNF production induced by the corresponding TLR ligand in the absence of MN-1 (shown by the dotted line as 100%). The data are pooled from 3 donors and a two tailed *t*-test of paired data was used with a 95% confidence interval to compare TNF release in the presence of 100 μM MN-1 compared to the same TLR ligand induced TNF in the absence of MN-1 (^*^p < 0.05). The analysis was performed individually for each TLR ligand. **(C)** Cell viability was assessed by a 3-[4,5 dimethylthiazol-2-yl]-2,5-diphenyl-tetrazolium bromide assay. Data are representative of 3 separate experiments performed in triplicate in 3 unrelated donors. Standard error of the mean was used for pooled experimental data whilst standard deviation was used in graphs showing representative experiments.

In our previous study, mianserin selectively inhibited the endosomal TLRs but not those expressed at the cell surface in primary human cells (10). To assess if this selectivity for endosomal TLR inhibition was retained by MN-1, primary human M-CSF macrophages were stimulated with Pam3 (TLR1/2), LPS (TLR4), and flagellin (TLR5) to activate cell surface TLRs and R-848 to activate TLR8 localized in the endosome. TNF production induced by the cell surface TLRs was not inhibited by MN-1 but a dose dependent inhibition of TLR8 induced TNF production was observed (Figure 3A). This was significant when data was pooled from 3 separate donors (Figure 3B). Cell viability was assessed using an MTT assay and found to be unaffected (Figure 3C).

### MN-1 Retained the Ability to Inhibit Spontaneous Release of TNF and IL-1 From Primary Human RA Synovial Membrane Cultures

**Figure 4:**
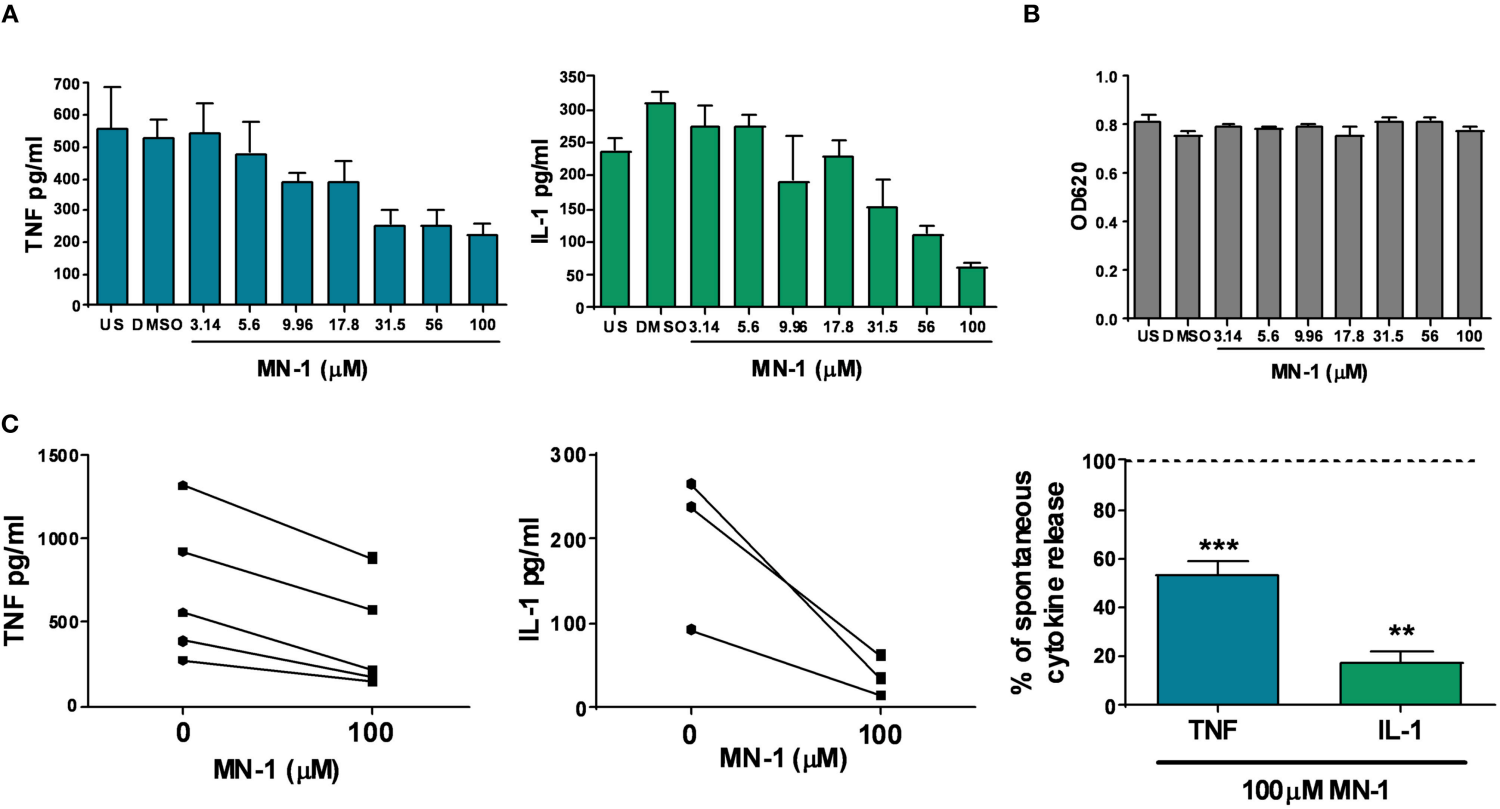
MN-1 inhibits spontaneous production of tumor necrosis factor (TNF) and interleukin (IL)-1 from human rheumatoid arthritis (RA) synovial membrane cultures. Human RA synovial membrane cultures were incubated for 24 h in the presence of media alone unstimulated (US) or media containing dimethyl sulfoxide (vehicle control) or MN-1. **(A)** A dose response was generated using a quarter log titration of MN-1. Data are representative of 3 independent experiments from independent donors. **(B)** Cell viability was assessed by a 3-[4,5 dimethylthiazol-2-yl]-2,5-diphenyl-tetrazolium bromide assay. Data are representative of 3 independent experiments from independent donors. **(C)** Pooled data is shown for 3–5 independent experiments from independent donors. All experiments were performed in triplicate with the average plotted from each donor. Where shown as a percentage, this was calculated from the maximal spontaneous release of TNF (*n* = 5) or IL-1 (*n* = 3) from untreated control cells. A two tailed *t*-test of paired data was used with a 95% confidence interval to compare cytokine release in the presence of 100 uM MN-1 compared to spontaneous cytokine release in the absence of MN-1 (^***^p < 0.001; ^**^p < 0.01) for the pooled data in part C. The analysis was performed for each cytokine separately. Standard error of the mean was used for pooled experimental data, whilst standard deviation was used in graphs showing representative experiments.

In addition to the ability to inhibit endosomal TLRs, we have previously published that mianserin could also inhibit spontaneous cytokine production in human rheumatoid synovial membrane cultures (10). These cultures spontaneously release cytokines and matrix metalloproteases without the need for exogenous stimulation and were used in the initial studies that identified anti-TNF as a potential therapeutic agent for rheumatoid arthritis (17). Incubation of MN-1 showed a dose dependent inhibition of the spontaneous release of TNF and IL-1 (Figure 4A). DMSO was used as a vehicle control at an equivalent amount to the 100 μM dose of MN-1. Cell viability was assessed using the MTT assay and found to be unaffected at all concentrations (Figure 4B). Data pooled from individual donors showed a significant inhibition of TNF by 46.6% ± 5.1 and IL-1 by 82.2% ± 4.2 in the MN-1 treated samples (Figure 4C).

## Discussion

Although many studies have highlighted the immunomodulatory properties of antidepressants and their ability to inhibit TLR signaling, the mechanism of action remains unclear. In this study, using structural derivatives of mianserin the results suggest that mianserin may modify TLR8 signaling independently of activity at 5-HT receptors and offers an insight into the structural requirements for the anti-inflammatory action of mianserin. Antidepressants have previously been suggested to be anti-inflammatory due to increasing the availability of 5-HT, which then activates monoamine receptors elevating cAMP and Ca^2+^ (13). Although inhibition of protein kinase A (PKA), a cAMP dependent kinase, has been shown to partially reverse the inhibitory effect of antidepressants on TLR4 in a microglial cell line, this was only by a modest 10–15%. In this same study, inhibiting adenylyl cyclase that converts ATP to cAMP, had no effect on the anti-inflammatory action of most antidepressants tested, furthermore addition of 5-HT to these cultures did not suppress TLR4 induced cytokine production (14). Similarly, in rheumatoid synovial membrane cultures where mianserin inhibits cytokine production, incubating these cultures with 5-HT also has no effect on cytokine production (7). Together these data suggest that although there may be a small contribution from PKA, elevation of cAMP is probably not the main mechanism by which antidepressants are immunomodulatory. Indeed, the concentrations of antidepressants required to inhibit cytokine production in primary human cells and rheumatoid synovial tissue is far in excess of those reported for 5-HT receptor binding (7, 10, 12).

To further investigate if 5-HT receptor binding was required for the anti-inflammatory action of mianserin, a series of structural derivatives were designed with the intention of decreasing the affinity for 5-HT receptor binding. The basic nitrogen is a key pharmacophoric component of 5-HT ligands and we designed a set of compounds that negated its effects either by (a) N-acylation (MN-1) or (b) tethering a polar carboxylic acid via an alkyl chain (MN-2 to 6). TLR8 was chosen for the primary screening in PBMCs, as this TLR produces a strong inflammatory cytokine response that was shown in our previous studies to be effectively inhibited by mianserin in primary human M-CSF derived macrophages. Furthermore, the spontaneous cytokine production from RA cultures that was also inhibited by mianserin was thought to be partially mediated through inhibition of TLR8; thus to test any successful derivatives in these culture it was important to check they could still inhibit TLR8 (10).

The first approach was the most successful with the acylated MN-1 showing the greatest inhibition of TLR8 signaling in the PBMC assay. As no inhibition was observed when using 80 μM of the compounds in almost all of the series tethering a polar carboxylic acid via an alkyl chain, with the exception of a very small decrease in the case of MN-3, this strategy was not pursued further. MN-1 however, did demonstrate a comparable inhibition of TLR8 induced TNF production in primary human PBMCs, with an improved EC_50_ compared to mianserin, despite having a significantly reduced affinity for 5-HT receptors. Comparable to mianserin, MN-1 also inhibited endosomal TLR8 but not the cell surface TLRs 1/2, 4, or 5 and significantly inhibited the spontaneous production of cytokines from the human RA synovial membrane cultures. The spontaneous TNF production was suppressed by an equivalent amount to that previously published for mianserin (10).

It can therefore be postulated that inhibition of TLR8-induced TNF and spontaneous cytokines from RA synovial cultures does not require the presence of a basic amine but is enhanced by the introduction of H-bond acceptors in this region of the molecule. This data also suggested that engagement of 5-HT receptors by mianserin may well be independent to its anti-inflammatory activity.

Mianserin has many known off target effects, however, most of the reported effects are at low nM concentrations. Thus, inhibition of endosomal TLRs remains a potential mechanism by which mianserin inhibits cytokine production in the RA synovial membrane cultures. Our previous study suggested that the anti-inflammatory target for mianserin may be at the receptor level or an early signaling event, as downstream activation of p38 mitogen activated protein kinase (MAPK) was inhibited by mianserin (10). Although, mianserin has been predicted using an *in silico* model to inhibit TLR4 signaling by binding to MD-2 (an accessory molecule involved in TLR4 activation by LPS), this is unlikely to be the mechanism for TLR8 inhibition, as TLR8 is not known to engage MD-2 (22). Furthermore, at the concentrations used in our study, mianserin does not inhibit TLR4 in primary human macrophages, whereas we have found that other antidepressants such as nortriptyline and amitriptyline can inhibit both TLR4 and TLR8 in human macrophages (data now shown) (10). Thus, there may be distinct mechanisms by which antidepressants can inhibit TLR4 and TLR8, with some antidepressants capable of inhibiting both.

Several events are required for the activation of endosomal TLRs. Firstly, molecular chaperones such as GP96 and Unc93B1 transport TLRs within the cell and in particular TLR8 to the endosomal compartment from where it can signal (23, 24). Then, before it can become activated, endosomal acidification, and cleavage by proteases are both necessary (25, 26). Accordingly, it is feasible that the mechanism of inhibition of TLR8 by mianserin and MN-1 may be through modulation of one or more of these processes.

Limitations of this study were that only one structural derivative of mianserin was explored in detail and that when testing MN-1 in the non-selective 5-HT receptor binding assay, this was performed at a single dose of 10 μM rather than generating a full dose response curve. It is possible that at a higher concentration MN-1 may have shown greater inhibition of 5-HT binding. However, the data indicate that MN-1 had a reduced affinity compared to mianserin and that fact that mianserin showed an almost complete inhibition of 5-HT receptor binding at 10 μM, yet was unable to inhibit TLR8 induced TNF at the same concentration supports the concept of the immunomodulatory activity being distinct from 5-HT receptor binding. It will now be important to develop a further series of structural analogs of MN-1 and to identify the target by which mianserin and MN-1 inhibit TLR8. However, demonstrating that 5-HT receptor binding may not be required for the anti-inflammatory mechanism of mianserin represents a significant incremental step toward understanding the structural requirements for the immunomodulatory properties of antidepressants.

## Data Availability

The datasets generated for this study are available on request to the corresponding author.

## Ethics Statement

This study was carried out in accordance with the recommendations of South East Coast—Brighton and Sussex research ethics committee and Brighton and Sussex Medical School Research Governance and Ethics Committee with written informed consent from all subjects. All subjects gave written informed consent in accordance with the Declaration of Helsinki. The protocol was approved by the South East Coast—Brighton and Sussex research ethics committee and Brighton and Sussex Medical School Research Governance and Ethics Committee.

## Author Contributions

SS participated in the design of the study, carried out the cell based experiments, analyzed the results, and drafted the manuscript. GC assisted with the mianserin inhibition assays in PBMCs. AJ-C, CL, and CT-S designed and commissioned synthesis of mianserin derivatives and contributed to discussions on the chemistry and pharmacology. All authors read and approved the final manuscript. This paper is dedicated to the memory of GC.

### Conflict of Interest Statement

SS has filed a patent application (WO/2008/090334). Use of 5HT Receptor Antagonists for Treating Arthritis. The remaining authors declare that the research was conducted in the absence of any commercial or financial relationships that could be construed as a potential conflict of interest.
