# Supplementary material for: Structural Modification of the Antidepressant Mianserin Suggests That Its Anti-inflammatory Activity May Be Independent of 5-Hydroxytryptamine Receptors
Source: Front Immunol. 2019 May 24;10:1167. doi: 10.3389/fimmu.2019.01167 (PMC6542943; doi:10.3389/fimmu.2019.01167)
Supplement: Supplementary file 1 [file Table_1.DOCX]

**Supplemental information**

**MN-1: Synthesis of compound 1-(1,3,4,14b-tetrahydrodibenzo[*c,f*]pyrazino[1,2-a]azepine-2 (10H)ethanone**

**Reaction Scheme:**

**Synthesis details:**

Step (A) Synthesis of ethyl 1, 3, 4, 14b-tetrahydrodibenzo[*c,f*]pyrazino[1,2-a] azepine-2 (10H)-carboxylate:

Mianserin (1.0 eq., 2.0 g, 7.56 mmol) was suspended in toluene (40 mL, 20 V) and ethyl chloroformate (20 eq., 16.4 g, 151.31 mmol) was added at RT. The reaction mixture was refluxed for 16 hrs. After completion (as monitored by TLC) the reaction mass was evaporated under vacuum to give 2.2 g of crude compound. The crude compound was purified by column chromatography using 60-120 mesh silica gel as stationary phase and the compound was eluted by using 5 % ethyl acetate as gradient in *n*-hexane to get 1.5 g of pure compound with 61.5 % Yield.

Step (B) Synthesis of 1,2, 3,4,10-14b-hexahydrodibenzo[c,f]pyrazino[1,2-a]azepine

Ethyl 1,3,4,14b-tetrahydrodibenzo[*c,f*]pyrazino[1,2-a]azepine-2-(10H)-carboxylate (1.0 eq., 1.5 g, 4.65 mmol) was dissolved in methanol (15 mL, 10 vol.), potassium hydroxide ( 4.8 eq., 1.25 g, 22.33 mmol) solution in water (11 mL) were added in the ester solution. The reaction mixture was refluxed 70 ^0^C for 8 hrs. The reaction was monitored by TLC. After completion of reaction (monitored by TLC), the reaction mass was neutralized with dilute HCl and extracted with ethyl acetate (2 x 15 mL). The ethyl acetate layer was separated and dried over sodium sulphate. Solvent was removed under vacuum to get 0.25 g of pure compound with 21.46 % yield.

Step (C) Synthesis of 1-(1,3,4,14b-tetrahydrodibenzo[*c,f*] pyrazino[1,2-a]azepine-2- (10H)ethanone:

1,2,3,4,10-14b-hexahydrodibenzo[c,f]pyrazino[1,2-a]azepine (1.0 eq., 0.235 g, 0.94 mmol) was dissolved in DCM (5.8 mL, 25 vol.). The reaction mixture was cooled down to 0 to 5 ^0^C and TEA (1.1 eq., 0.104 g, 1.03 mmol) and acetyl chloride (1.1 eq., 0.081 g, 1.03 mmol) were added in the reaction solution. The reaction mixture was allowed to attain RT and stirred for 2-3 hrs at RT. After completion (monitored by TLC) of reaction, the reaction mass was poured in to ice water (5 mL) and the product was extracted with DCM (2x 5 mL). The combined organic layer was dried over sodium sulphate and the volatiles were removed under vacuum to obtain 0.25 g of crude compound. The crude compound was purified by column chromatography using 60-120 mesh silica gel as stationary phase and the compound was eluted by using 26 % ethyl acetate as gradient in *n*-hexane to afford 0.11 g of pure compound with 40.14 % Yield.

Analytical data

| Compound  ID | HPLC  Purity | MS (M^+^) | ^1^H NMR spectrum was recorded on a Bruker 400 |
| --- | --- | --- | --- |
| MN-1 | 96.88 % | 293 | ^1^H NMR(CDCl_3_) δ 6.91-7.20 (m,8H,-ArH),4.82-4.85 (d,1H,-Ar-CH_2_-Ar), 4.62-4.77 (d,1H,-Ar-CH_2_-Ar), 3.91-3.97 (t,1H,-N-CH_2_-Ar), 3.75-3.77 (t,2H,-N-CH_2_-); 3.45-3.51 (t,1H,-N-CH_2_),3.36-3.39 (d,2H,-N-CH_2_-), 3.20-3.28 (m,1H,-N-CH), 2.11-2.21 (s,3H,-CH_3_-C=O). |

**MN-2: Synthesis of compound 2-(1,3,4,14b-tetrahydrodibenzo[*c,f*]pyrazino[1,2-a]azepine-2 (10H)acetic acid**

**Reaction Scheme**

**Step-(C): ethyl 2-(1,2,3,4,10-14b-hexahydrodibenzo[*c,f*]pyrazino[1,2-a]azepine-2(10H)-yl)acetate

STEP-C

1,2,3,4,10-14b-hexahydrodibenzo[c,f]pyrazino[1,2-a]azepine (1.0 eq., 1.0 g, 4.0 mmol) was dissolved in acetonitrile (35 mL, 35 vol) and potassium carbonate (1.1 eq., 0.607 g, 4.4 mmol) was added in to the solution. The reaction mixture was heated at 80^°^C and ethyl-2-bromo-acetate (1.1 eq., 0.730 g, 4.4 mmol ) was added in to reaction mass and heating continued at 80^°^C for 16 hrs. After completion (monitored by TLC) the reaction was cooled down to RT and filtered through celite bed. The filtrate was concentrated under vacuum to give 2.1 g crude compound as yellow oil. The crude compound was purified by using column chromatography using 60-120 mesh silica gel as stationary phase and the compound was eluted by using 10 % ethyl acetate as gradient in *n*-hexane to get 1.05 g of pure product with 78.13 % Yield.

Step (D) Synthesis of 2-(1,2,3,4,10-14b-hexahydrodibenzo[*c,f*]pyrazino[1,2-a] azepine-2(10H)-yl)acetic acid:

STEP-D

Ethyl 2-(1,2,3,4,10-14b-hexahydrodibenzo[*c,f*]pyrazino[1,2-a]azepine-2(10H)-yl) acetate (1.0 eq., 1.05 g, 3.12 mmol) was disslved in methanol (30 mL, 30 vol) and a solution of sodium hydroxide ( 2.0 eq., 0.25 g, 6.24 mmol) in water (6.2 mL) was added in it. The reaction mixture was heated at 70 ^°^C for 2 h. After completion (monitored by TLC) the reaction mass was concentrated and water (10 mL) was added in it. The aqueous solution was neutralized with 1M HCl to obtain white precipitates. The solid was filtered and dried under vacuum to get 0.3 g of compound MN-2 with 29.77 % yield.

Analytical data

| Compound  ID | HPLC  Purity | MS (M^+^) | ^1^H NMR spectrum was recorded on a Bruker 400 |
| --- | --- | --- | --- |
| MN-2 | 98.95 % | 308.5 | ^1^H NMR (MeOD) δ 7.07-7.25 (m,7H,-ArH), 6.92-6.95 (m,1H,-ArH), 4.80-4.83(d,1H,-Ar-CH_2_-Ar), 4.34-4.37 (d,1H,-Ar-CH_2_Ar), 3.81-3.84 (d,1H,-N-CH-Ar), 3.73 (d,2H,-N-CH_2_), 3.61-3.64 (t,2H,-N-CH_2_-CH_2_), 3.52-3.55 (t,1H,-N-CH2-), 3.38-3.43 (t,2H,-N-CH_2_-C=O), 3.30-3.35 (t,1H,-N-CH2-), |

**MN-3: Synthesis 3-(1,2,3,4,10-14b-hexahydrodibenzo[*c,f*]pyrazino[1,2-a]azepine-2(10H)-yl)proponic acid**

**Reaction Scheme**

Step (C) Synthesis of ethyl 3-(1,2,3,4,10-14b-hexahydrodibenzo[*c,f*]pyrazino[1,2-a]azepine-2(10H)-yl)propanote:

1,2,3,4,10-14b-hexahydrodibenzo[c,f]pyrazino[1,2-a]azepine (1.0 eq., 1.0 g, 4.0 mmol) was dissolved in ACN (40 mL, 40 vol.), Potassium carbonate ( 1.1 eq., 0.607 g, 4.4 mmol) and Ethyl-2-bromo propionate (1.1 eq, 0.79 g, 4.4 mmol) were added in it. The reaction mass was refluxed for 8-10 hrs. After completion of reaction (monitored by TLC) the reaction mass was allowed to cooled to RT and filtered through celite bed. The filtrate was evaporated under vacuum to obtain 0.9 g of compound with 64.33 % Yield. The isolated compound was used as such for the next step.

Step (D) Synthesis 3-(1,2,3,4,10-14b-hexahydrodibenzo[*c,f*]pyrazino[1,2-a]azepine-2(10H)-yl)proponic acid:

Ethyl 3-(1,2,3,4,10-14b-hexahydrodibenzo[*c,f*]pyrazino[1,2-a]azepine-2(10H)-yl)propanote (1.0 eq., 0.9 g, 2.57 mmol) was dissolved in methanol (27 mL, 30 vol.) and sodium hydroxide ( 2.0 eq., 0.205 g, 5.14 mmol) dissolved in water (5 mL) was added in the solution. The reaction mixture was heated at 70-80 ^0^C for 2 hrs. After completion of reaction (monitored by TLC), the reaction mass was allowed to cool down to RT and poured in ice water (50 mL) and neutralized with dilute hydrochloric acid. The precipitated solid was filtered and dried under vacuum to get 0.228 g of pure compound with 27.56 % Yield.

Analytical data

| Compound  ID | HPLC  Purity | MS (M^+^) | ^1^H NMR spectrum was recorded on a Bruker 400 |
| --- | --- | --- | --- |
| MN-3 | 97.13 % | 323 | ^1^H NMR (MeOD) δ 7.09-7.26 (m,7H,-ArH), 6.95-6.97 (m,1H,-ArH), 4.84-4.83 (dd,1.5H,-Ar-CH_2_-Ar, & Ar-CH-N), 4.25-4.33 (m,1.5H,-Ar-CH_2_-Ar, & Ar-CH-N), 3.69-3.807(d,1H,-Ar-CH_2_-Ar), 3.52-3.60 (m,4H,-N-CH_2_-C), 3.42-3.52 (t,1H,-N-CH_2_-C and & -N-CH_2_-CO ), 2.89-2.93 (t,1H,-N-CH_2_-C=O) |

**MN-4: Synthesis of 2-(1,2,3,4,10-14b-hexahydrodibenzo[*c,f*]pyrazino[1,2-a]azepine-2(10H)-yl)butanoic acid:**

**Reaction Scheme**

Step (C) Synthesis of ethyl 2-(1,2,3,4,10-14b-hexahydrodibenzo[*c,f*]pyrazino [1,2-a]azepine-2(10H)-yl)butanoate:

1,2,3,4,10-14b-hexahydrodibenzo[c,f]pyrazino[1,2-a]azepine (1.0 eq., 1.0 g, 4.0 mmol) was dissolved in acetonitrile (40 mL, 40 vol.) and potassium carbonate (1.1 eq., 0.607 g, 4.4 mmol) was added in to the solution. The reaction mixture was heated at 80 °C and ethyl-4-bromo-butanoate (1.1 eq., 0.858 g, 4.4 mmol) was added in to reaction mass and continued at 80 ^°^C for 16 h. After completion (monitored by TLC) the reaction was cooled down to RT and filtered through celite bed. The filtrate was concentrated under vacuum to obtain crude compound as yellow oil (1.4 g) with yield 96.16%. The crude compound was used as such for the next step.

Step (D) Synthesis of 2-(1,2,3,4,10-14b-hexahydrodibenzo[*c,f*]pyrazino[1,2-a] azepine-2(10H)-yl)butanoic acid:

Ethyl 2-(1,2,3,4,10-14b-hexahydrodibenzo[*c,f*]pyrazino[1,2-a]azepine-2(10H)-yl) butanoate (1.0 eq., 1.4 g, 3.84 mmol) was dissolved in methanol (42 mL, 30 vol.) and a solution of sodium hydroxide( 2.0 eq., 0.307 g, 7.68 mmol) in water (3 mL) was added in it. The reaction mixture was heated at 70 ^°^C for 2 h. After completion (monitored by TLC) the reaction mass was poured in ice-water (50 mL) and neutralized it with 1M HCl to obtain white precipitates. The solid was filtered and dried under vacuum to obtain 0.38 g of compound MN-4 with 29.4 % yield.

Analytical data

| Compound  ID | HPLC  Purity | MS (M^+^) | ^1^H NMR spectrum was recorded on a Bruker 400 |
| --- | --- | --- | --- |
| MN-4 | 94.18 % | 337 | ^1^H NMR (CDCI_3_) δ 7.11-7.17 (m,6H,-ArH), 6.99-7.01 (d,1H,-ArH); 6.93-6.95 (m,1H,-ArH); 4.70-4.73 (d,1H,-Ar-CH_2_-Ar), 4.42-4.44 (dd,1H,-N-CH-C), 3.69-3.72 (d+t,2H,-N-CH_2_-CH_2_ , & Ar-CH_2_-Ar); 3.47 (d,1H,-N-CH_2_-C), 3.47 (t,1H,-N-CH-Ar), 3.33-3.36(d,1H, ,-N-CH_2_-C),3.07-3.11 (t,2H,N-CH_2_-CH_2_), 2.91-297(t,2H,-N-CH_2_-CH_2_), 2.52-2.56 (t,2H,-CH_2_-CH_2_-C=O-), 2.05-2.08 (m,2H,-CH_2_-CH_2_-CH_2_-) |

**MN-5: Synthesis of Ethyl 2-(1,3,4,-14b-tetrahydrodibenzo[c,f]pyrazino[1,2-a]azepine-2(10H)propanoic acid:**

**Reaction Scheme**

Step (C) Synthesis of Ethyl 2-(1,3,4, -14b-tetrahydrodibenzo[c,f]pyrazino[1,2-a]azepine-2(10H)propainotate:

1,2,3,4,10-14b-hexahydrodibenzo[c,f] pyrazino[1,2-a]azepine (1.0 eq., 0.7 g, 2.8 mmol) was dissolved in in acetonitrile (28 mL, 40 vol.), potassium carbonate ( 1.1 eq., 0.425 g, 3.08 mmol) and ethyl-2-bromopropanoate (1.1 eq., 0.506 g, 3.08 mmol) were added. The reaction mixture was heated for 8-10 h. After completion of reaction (monitored by TLC), the reaction mass was cooled down to RT and filtered through celite bed. The filtrate was evaporated under vacuum to obtain 0.9 g of compound with 91.9 % Yield. The isolated compound was used as such for the next step.

Step (D) Synthesis of Ethyl 2-(1,3,4,-14b-tetrahydrodibenzo[c,f]pyrazino[1,2-a]azepine-2(10H)propanoic acid:

Ethyl 2-(1,3,4,-14b-tetrahydrodibenzo[c,f]pyrazino[1,2-a]azepine-2(10H)propinotate(1.0 eq., 0.9 g, 2.57 mmol) was dissolved in methanol (27 mL, 30 vol.) and sodium hydroxide solution ( 2.0 eq., 0.206 g, 5.14 mmol) in water (5 mL) added. The reaction mixture was heated at 70-80 ^°^C for 2 h. After completion of reaction (monitored by TLC) the reaction mass was cooled down to RT and poured in Ice water (50 mL). The solution was neutralized with dilute hydrochloric acid and the precipitated solid compound was filtered and dried under vacuum to furnish 0.32 g of pure compound with 38.9 % Yield.

Analytical data

| Compound  ID | HPLC  Purity | MS (M^+^) | ^1^H NMR spectrum was recorded on a Bruker 400 |
| --- | --- | --- | --- |
| MN-5 | 98.86% | 323 | ^1^H NMR (MeOD) δ 7.07-7.21 (m,7H,-ArH); 6.92-6.94 (t,1H,-ArH); 4.80-4.83 (d,1H,-Ar-CH_2_-Ar), 4.29-4.33 (d,1H,-N-CH-AR), 3.49-3.65 (m,4H,-N-CH_2_-CH_2_ & N-CH_2_- CH_2_; ,-Ar-CH_2_-Ar), 3.37-3.41(m,3H,-N-CH_2_-CH_2_), 3.32-3.25 (q,1H,-N-CH-CH_3_)), 1.50-1.56 (d,3H,-CH_3_-CH-N) |

**2-methyl-4-(1,3,4, -14b-tetrahydrodibenzo[c,f] pyrazino[1,2-a]azepine-2(10H) butanoic acid:**

**Reaction Scheme**

Step (C) Synthesis of Methyl 2-methyl-4-(1,3,4-14b-tetrahydrodibenzo[c,f]pyrazino [1,2-a]azepine-2(10H)butanoate:

*N-*

1,2,3,4,10-14b-hexahydrodibenzo[c,f]pyrazino[1,2-a]azepine (1.0 eq., 0.65 g, 2.6 mmol) was dissolved in acetonitrile (26 mL, 40 vol.), potassium carbonate ( 1.1 eq., 0.395 g, 2.86 mmol) and methyl-4-chloro-2-methyl butanoate (1.1 eq., 0.431 g, 2.86 mmol) were added. The reaction mixture was refluxed for 8-10 h. After completion of reaction (monitored by TLC) the reaction mass was cooled down to RT and filtered through celite bed. The filtrate was evaporated under vacuum to obtain 0.5 gm of compound with 52.8 % Yield. The compound was used as such for the next step.

Step (D) Synthesis of 2-methyl-4-(1,3,4, -14b-tetrahydrodibenzo[c,f] pyrazino[1,2-a] azepine-2(10H) butanoic acid::

Methyl 2-methyl-4-(1,3,4-14b-tetrahydrodibenzo[c,f]pyrazino[1,2-a]azepine-2(10H) butanoate (1.0 eq., 0.5 g, 1.37 mmol) was dissolved in methanol (15 mL, 30 vol.) and sodium hydroxide solution ( 2.0 eq., 0.109 g, 2.74 mmol) in water (3 mL) was added. The reaction mixture was refluxed at 70-80 ^°^C for 2 h. After completion of reaction (monitored by TLC) the reaction mass was cooled down to RT and poured the reaction mixture in Ice water (50 mL) and neutralized with dilute hydrochloric acid. The precipitated solid compound was filtered and dried under vacuum to obtain 0.124 g of pure compound MN-6 with 25.8 % Yield.

Analytical data

| Compound  ID | HPLC  Purity | MS (M^+^) | ^1^H NMR spectrum was recorded on a Bruker 400 |
| --- | --- | --- | --- |
| MN-6 | 99.00 % | 351 | ^1^H NMR (MeOD) δ 7.08-7.19 (m, 7H,-ArH); 6.92-6.96 (m,1H,-ArH); 4.80-4.83 (d,1H,Ar-CH2-Ar), 3.78-3.81 (d,1H,-N-CH-ArC), 3.54-3.63 (m,3H,-N-CH2-C, Ar-CH2-Ar); 3.41-3.44 (m,2H,-N-CH_2_-CH_2_), 3.31-3.33 (m,2H,-N-CH-CH_3_)), 2.25-2.26 (m, 1H, ,-OC-CH-CH_2_); 2.1-2.2 (m, 2H, ,-CH-CH_2_-CH_2_) -1.91-2.01 (m, 1H, C-CH-CH_3_); 1.28-1.31 (d,3H,-CH_3_-CH-N). |
